# Genomic insights into bacterial communities of coenocytic algae using metagenome-assembled genomes

**DOI:** 10.3389/fmicb.2026.1851259

**Published:** 2026-10-06

**Authors:** Gerardo Laureano, Xochitl Ramirez, Ashley Scoles, Christina Johne, Coralys M. Colón Sáez, Yerlianys M. Hernández Ortiz, Jennifer Soleyman, Ramón E. Rivera-Vicéns, Alok Arun

**Affiliations:** 1 Department of Sciences and Technology, Inter American University of Puerto Rico, Barranquitas, PR, United States; 2 Dr. James Youngblom Center for Sustainable Biotechnology, Department of Biological Sciences, California State University Stanislaus, Turlock, CA, United States

**Keywords:** algae, Bryopsidales, coenocytic, MAGS, *Vaucheria*

## Abstract

Coenocytic algae are organisms that undergo karyokinesis without cytokinesis, resulting in multinucleated cells. Most research on bacterial communities in coenocytic algae has used 16S rRNA sequencing, primarily focusing on the order Bryopsidales of green coenocytic algae. Recent studies have analyzed metagenome-assembled genomes (MAGs) from algal hosts across multiple taxa, such as Chlorophyta, Phaeophyta, and Rhodophyta, revealing further insights into bacterial biosynthetic machinery and potential symbiotic relationships. Given the cosmopolitan distribution of coenocytic algae, such as *Bryopsis*, *Caulerpa*, *Codium*, and the yellow-green alga *Vaucheria*, and their unique morphology, there is a need to better understand their associated bacterial communities. To address this, filaments of *Vaucheria bursata* LB2067 were sequenced using the Illumina NovaSeq instrument, and all publicly available short- and long-read datasets from coenocytic algae were screened for MAG recovery. All recovered MAGs from both Bryopsidales and Vaucheriales showed a high dominance of Pseudomonadota at the phylum level, but no consistent patterns at the order or family levels. Core metabolic pathways, including glycolysis, the TCA cycle, and amino acid biosynthesis, were present across MAGs from both coenocytic and non-coenocytic algae. Pathways with significantly higher completeness in coenocytic-associated MAGs included protein secretion (Sec-SRP), which showed the strongest effect, motility (flagellar assembly), thiamin biosynthesis, and polyhydroxybutyrate (PHB) synthesis (Mann–Whitney *U*, *p*adj < 0.05). Notably, N-acetylglutaminylglutamine amide (NAGGN) biosynthetic gene clusters (BGCs) were found only in MAGs from coenocytic orders, whereas polysaccharide utilization loci (PULs) were present in all MAGs analyzed. These results indicate that bacterial communities associated with coenocytic algae are complex, with diverse survival strategies adapted to challenging and variable environments.

## Introduction

Eukaryotic organisms harbor specific microbiomes that can perform vital functions for the host, such as nutrient provision and protection against pathogens (Compant et al., 2019; Peixoto et al., 2021). A microbiome is a microbial community that occupies a well-defined habitat or host and performs distinct physicochemical functions (Vila Duplá, 2025). The composition of a microbiome can include a wide range of microorganisms, such as bacteria, archaea, algae, and fungi. Microbiomes in aquatic environments can influence the host’s health and adaptation to environmental stress. This is significant in algae, given their symbiotic relationship with their associated bacterial communities (Van Der Loos et al., 2019).

The majority of research on the composition of algal bacterial communities has focused on sequencing specific regions of the 16S rRNA gene (Větrovský and Baldrian, 2013; Singh and Reddy, 2014). This approach is widely used because the 16S rRNA gene is highly conserved, ubiquitous in bacteria, and evolves slowly, making it ideal for taxonomic identification. However, when the targeted region lacks sufficient sequence variation in the 16S rRNA region, identification may fail (Lamoureux et al., 2022). A key limitation is that 16S rRNA sequencing is primarily used for taxonomic prediction and diversity analysis (Mathieu et al., 2023). It cannot provide detailed information on metabolic interactions between algae and bacteria or reveal all interactions or pathways.

In contrast to 16S rRNA sequencing, whole-genome sequencing (WGS) of the algal host enables the recovery of metagenome-assembled genomes (MAGs). This approach is increasingly used to investigate both the metabolic interactions and taxonomic identification of algal microbiomes (Meziti et al., 2021; Mirete et al., 2025). Such metabolic exchanges include algal carbon cycling, where heterotrophic bacteria, such as those in the phylum Bacteroidota, possess numerous polysaccharide-degrading enzymes and polysaccharide utilization loci (PULs) (Martens et al., 2011; Lu et al., 2023). Enzymes encoded in these gene clusters enable the microbiome to degrade host polysaccharides such as alginate, fucoidan, agar, and carrageenan (Grondin et al., 2017; Feng et al., 2022; Gu et al., 2023; Lu et al., 2023; Zhang et al., 2024). This degradation provides the bacterial community with essential building blocks for the production of metabolites, such as vitamin B12 (Balabanova et al., 2021). In contrast to PULs, biosynthetic gene clusters (BGCs) are groups of genes that encode different enzymatic machinery required to produce specific metabolites (Covington et al., 2021). BGCs are critical for many organisms, particularly bacteria, as these secondary metabolites underpin essential pharmaceuticals, agrochemicals, and ecological mediators (Blin et al., 2023, 2025). Identification of BGCs and PULs enables the analysis of metabolic capabilities that cannot be determined from 16S rRNA alone.

In recent years, macroalgal bacterial communities have been recovered using metagenome-assembled genomes (MAGs) from diverse taxa, including Phaeophyceae, Rhodophyceae, and Chlorophyceae, from the genera *Nereocystis, Saccharina, Pyropia, and Ulva*, respectively. Metabolic reconstruction of these MAGs has revealed their core functional potential, particularly in carbohydrate metabolism and the degradation of host structural polysaccharides, as indicated by their high representation (Wang et al., 2022; Weigel et al., 2022; Lu et al., 2023; Zhang et al., 2024). Additionally, these studies suggest that macroalgal-associated bacteria have the capacity to produce essential bioactive compounds and secondary metabolites, via BGCs involved in the synthesis of vitamins and phytohormones such as auxin, as well as enzymes like ACC deaminase that may help regulate host stress responses (Wang et al., 2022; Weigel et al., 2022; Lu et al., 2023).

Bacterial communities associated with yellow-green algae (Xanthophyceae), a distinct algal group characterized by xanthophyll pigments, remain poorly explored. Xanthophyceae exhibit an exceptional range of morphologies, including unicellular forms, coenocytic filaments, simple multicellular thalli, and complex multicellular thalli. Coenocytic organisms consist of multinucleate cells that lack internal cross-walls (septa), forming a continuous cytoplasm. Despite this remarkable morphological diversity, relatively little is known about the composition of their associated bacterial communities or the metabolic interactions that may differ across these growth forms. A recent study (Ochiai et al., 2025) reconstructed MAGs from the coenocytic alga *Bryopsis* sp. KO-2023 using both long and short-read sequencing, reporting a dominance of Alphaproteobacteria and Bacteroidota. Apart from this work, most studies of bacterial communities associated with coenocytic algae have relied primarily on 16S rRNA sequencing.

Coenocytic algae represent a unique morphological organization in which repeated nuclear divisions (karyokinesis) occur without cytokinesis, resulting in a multinucleate organism composed of a continuous cytoplasm lacking internal cross-walls. This morphology has evolved independently in several algal lineages, including the green algal genera *Caulerpa* and *Bryopsis* and the yellow-green algal genus *Vaucheria*, all of which have broad, cosmopolitan distributions. Because coenocytic algae possess a continuous cellular architecture distinct from both unicellular and multicellular forms, they provide an excellent model for investigating how algal morphology influences the composition, spatial organization, and metabolic interactions of associated bacterial communities. Analysis of bacterial communities of coenocytic algae has been dominated by 16S rRNA amplicon sequencing in *Caulerpa*, *Bryopsis,* and *Vaucheria*, revealing bacterial communities composed of Proteobacteria, Bacteroidetes, and Planctomycetes (Devine et al., 2012; Aires et al., 2015; Roth-Schulze et al., 2018; Liang et al., 2019; Kopprio et al., 2021; Van Duijnhoven et al., 2024; Ochiai et al., 2025; Delbridge et al., 2004).

To date, the bacterial community composition of *Vaucheria* has only been reported for *Vaucheria litorea* using 16S rRNA sequencing (Devine et al., 2012). A deeper understanding of both the taxonomic composition and metabolic interactions of bacterial communities associated with *Vaucheria* species could provide valuable insights into the relationship between algal morphology and bacterial community composition. *Vaucheria* belongs to the class Xanthophyceae and is taxonomically distinct from coenocytic green algae such as *Bryopsis* and *Caulerpa* (Chlorophyta). This study aims to investigate potential associations between bacterial communities and coenocytic algal morphology through a comprehensive analysis of recovered metagenome-assembled genomes (MAGs), with a particular focus on identifying conserved metabolic functions associated with the surfaces of these giant single-celled algae. To this end, vegetative filaments of *Vaucheria bursata* LB2067 were sequenced using the Illumina sequencing platform. In addition, recovered MAGs were compared with those obtained from 11 publicly available whole-genome sequencing (WGS) datasets from various other coenocytic algae.

## Materials and methods

### Algae datasets used in this study

The following genera of coenocytic algae were selected based on the availability of sequencing data in the NCBI SRA database accessed in December 2025: *Ostreobium quekettii, Derbesia tenuissima, Derbesia marina, Codium adhaerens, Caulerpa lentillifera, Caulerpa ashmeadii, Avrainvillea amadelpha, Penicillus capitatus, Penicillus lamourouxii, Caulerpa prolifera* and *Vaucheria bursata* (Table 1). Previously reported MAGs from the genus *Bryopsis* were also included in the analysis (Ochiai et al., 2025).

**Table 1:** Summary statistics of MAGs recovered from WGS reads of coenocytic algae, classified by quality as near complete, high quality (HQ), good quality (GQ), and low quality (LQ), and by read type as long reads (LR), short reads (SR), or hybrid.

Organism	Taxonomic order	Bioproject	Total MAGs	Near complete	HQ	GQ	LQ	LR	SR	Hybrid
*Vaucheria bursata LB2067*	Vaucheriales	PRJNA1020787	11	0	10	1	0	0	11	0
*Vaucheria bursata*	Vaucheriales	PRJNA894593	30	0	22	8	0	0	30	0
*Ostreobium **quekettii***	Bryopsidales	PRJEB39309	37	0	34	3	0	36	0	1
*Derbesia tenuissima*	Bryopsidales	PRJNA924561	64	0	43	16	5	0	64	0
*Derbesia marina*	Bryopsidales	PRJNA924561	41	0	37	4	0	0	41	0
*Codium adhaerens*	Bryopsidales	PRJNA924561	34	0	28	5	1	0	34	0
*Caulerpa lentillifera*	Bryopsidales	PRJDB5734	4	0	2	2	0	3	1	0
*Caulerpa ashmeadii*	Bryopsidales	PRJNA515488	6	0	6	0	0	0	6	0
*Avrainvillea amadelpha*	Bryopsidales	PRJNA924561	30	0	17	13	0	0	30	0
*Penicillus capitatus*	Bryopsidales	PRJEB87011	61	16	32	5	8	61	0	0
*Penicillus lamourouxii*	Bryopsidales	PRJEB88235	**19**	**4**	**8**	**2**	**5**	19	0	0
*Caulerpa prolifera*	Bryopsidales	PRJEB94584	7	1	5	0	1	7	0	0

### Culture conditions, DNA extraction, and sequencing

*Vaucheria bursata* LB2067 cultures were obtained from the UTEX Culture Collection of Algae at UT-Austin (Austin, USA). The cultures were maintained under a 16:8 light/dark photocycle at 25 °C. Isolation of genomic DNA from filaments was performed using the NucleoSpin Plant II kit (Macherey-Nagel), according to the manufacturer’s instructions. Purified genomic DNA was used for library construction following a standard Illumina library preparation protocol. Briefly, DNA was fragmented and subjected to end repair, adenylation of 3′ ends, adapter ligation, and PCR amplification to generate indexed sequencing libraries. Library concentration and insert-size distribution were evaluated prior to sequencing. Indexed libraries were pooled and sequenced on an Illumina NovaSeq instrument to produce 150-bp paired-end reads.

### Metagenome assembly and binning

Different strategies for metagenome-assembled genome (MAG) recovery were applied according to the data availability for each alga: short-read (SR), long-read (LR), or hybrid (SR + LR). For datasets consisting solely of Illumina short reads (SR), the metaWRAP pipeline (v1.3) was used (Uritskiy et al., 2018), specifically the Read_QC, Assembly with MegaHit v1.1.4 (Li et al., 2015), Binning, Bin_Refinement, and Reassembly_Bin modules. Illumina datasets comprising triplicate runs from the same biological sample were concatenated before MAG recovery (Supplementary Table S1). Bins with a total size exceeding 10 Mbp were removed to filter out potential eukaryotic contamination or assembly artifacts. All software packages were used with default parameters unless otherwise specified. Host DNA was not removed prior to binning to support a separate, ongoing study. For datasets consisting only of long-read (LR) data (PacBio or Nanopore), the following pipeline was used: raw long reads were assembled using Flye (v2.9.6) (Freire et al., 2022) with the --meta option. Depending on the sequencing platform, the --pacbio-raw, --pacbio-hifi, or --nano-raw flag was applied. Raw long reads were mapped to the resulting assemblies using minimap2 (v2.30) (Li, 2018) with the appropriate preset (map-pb, map-hifi, or map-ont). Coverage depth across contigs was calculated using the jgi_summarize_bam_contig_depths script from MetaBAT2 (v2.18) (Kang et al., 2019). Both MaxBin2 (v2.2.7) (Wu et al., 2016) and MetaBAT2 were implemented for the binning process. The final bin set was produced by de-replicating and consolidating bins from both MaxBin2 and MetaBAT2 using DAS Tool (v1.1.7) (Sieber et al., 2018) (Supplementary Figure S1).

In cases where datasets were available for both LR and SR (i.e., hybrid), the processes described above were performed independently for each data type. To identify potentially identical bins between the SR and LR bin sets, FastANI (v1.34) (Jain et al., 2018) was implemented with a 95% threshold, and the resulting pairs were processed in parallel. The raw reads were mapped using minimap2 (v2.30) for LR and Bowtie 2 (v2.5.4) (Langmead and Salzberg, 2012) for SR to the bins identified as identical by FastANI. The corresponding mapping files were converted to BAM format, and mapped FASTQ files were extracted using SAMtools (v1.22.1) (Li et al., 2009). Reassembly was performed using Unicycler (v0.5.1) (Wick et al., 2017), and the quality of the reassembled bins was compared with that of the original bin pairs in terms of contamination, completeness, genome size, N50, and contig count using CheckM2 (v1.1.0) (Chklovski et al., 2023) (Supplementary Figure S1). The highest-quality bins were selected for the final bin set.

### MAG quality assessment, de-replication, and taxonomic classification

The quality of the final MAGs was assessed using CheckM2 (v1.1.0), which was used to estimate genome completeness and contamination. Based on these estimates, MAGs were classified as near complete, high quality (HQ), good quality (GQ), or low quality (LQ) following Almeida et al. (2019). HQ MAGs had >90% completeness and ≤5% contamination, GQ MAGs had ≥50 and <90% completeness and ≤5% contamination, and LQ MAGs had <50% completeness and ≤10% contamination. Bins that exceeded the contamination threshold for their respective completeness category were discarded and are not reported. HQ MAGs recovered as a single contig were reported separately as near complete. MAGs with ≥10% contamination were excluded from subsequent analyses regardless of completeness; this criterion resulted in the removal of one MAG. De-replication of MAGs was performed to remove replicated genomes from the dataset by implementing Galah (v0.4.2)^1^ for clustering. The CheckM2 output was then used to manually select the best representative genome from each cluster based on contamination, completeness, genome size, N50, and contig count.

Taxonomic classifications were conducted primarily using GTDB-Tk (2.6.1 + galaxy0) (Genome Taxonomy Database Toolkit) (Chaumeil et al., 2022) using the database version R226. Using classifications derived from GTDB-Tk, taxonomic lineages were assigned. Barrnap (v0.9)^2^ was used to identify all rRNA sequences present in MAGs. QIIME 2 (v2025.10) (Bolyen et al., 2019) was used with the SILVA 138.2 database (Quast et al., 2012) for taxonomic prediction of the extracted 16S sequences to validate the genus- and species- GTDB-Tk assignments. For this, only SILVA similarity scores of equal to or greater than 94.5 and 98.7% were used for genus and species, respectively.

### Structural and functional analyses and BGC identification

Structural annotation was performed using Bakta (v1.9.11) (Schwengers et al., 2021) with the --metagenome option. For comprehensive functional annotation, genes were processed using eggNOG-mapper (v2.1.13) (Cantalapiedra et al., 2021). KEGGaNOG (v1.1.19)^3^ was implemented to evaluate the completeness of KEGG pathways present in each MAG using the output from eggNOG-mapper. Pathways detected in all MAGs were excluded from Figure 1, such that only pathways showing variation in detection across the MAG set are displayed. This filtering criterion was based on pathway detection rather than completeness; therefore, a pathway was retained if it was undetected in at least one MAG, regardless of its completeness in the remaining MAGs. In addition, AntiSMASH (v8.0.4) (Blin et al., 2025) was used to identify biosynthetic gene clusters (BGCs), and BiG-SCAPE (v2.0.0)^4^ was used to classify and count BGC families present throughout the algal community (Supplementary Figure S2).

**Figure 1:**
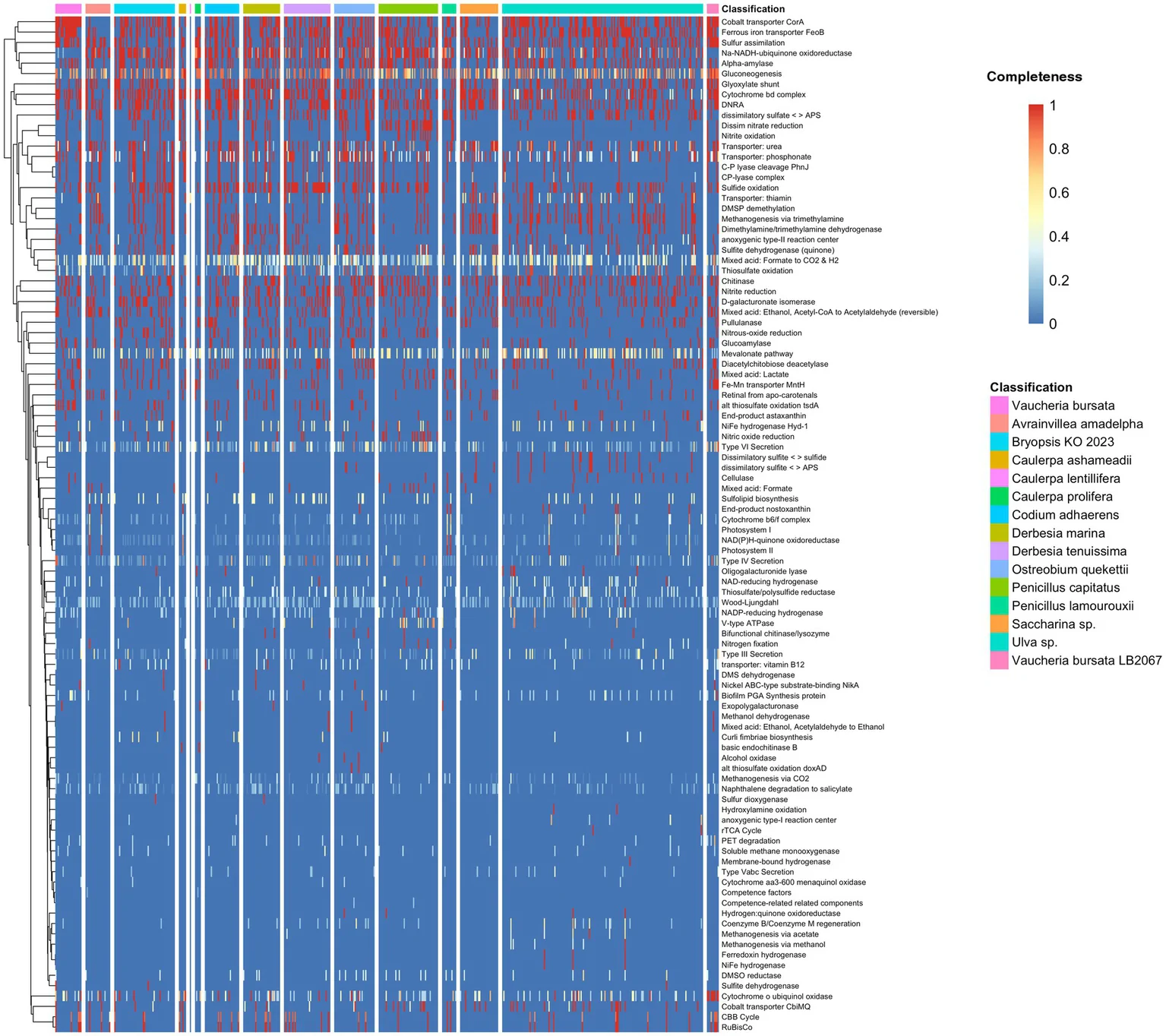
Metabolic functional profiles of bacterial metagenome-assembled genomes (MAGs) associated with coenocytic and non-coenocytic algae. The heatmap shows KEGG pathway completeness across a non-redundant set of 512 MAGs, comprising 311 MAGs from coenocytic algal hosts and 201 from non-coenocytic hosts. Genes were annotated using eggNOG-mapper v2.1.13, and KEGG pathway completeness was calculated with KEGGaNOG v1.1.19. Pathways detected in all MAGs were excluded, such that the heatmap displays only pathways with variable presence or completeness across the MAG set. Each column represents one MAG, and each row represents one KEGG pathway. Pathway completeness ranges from 0 (absent; blue) through intermediate completeness (pale yellow) to 1 (complete; red), as indicated by the color scale. The annotation bar above the heatmap identifies the algal host species from which each MAG was recovered, with colors corresponding to the classification legend. MAGs (columns) are grouped by host species, whereas KEGG pathways (rows) are ordered by hierarchical clustering, with the resulting dendrogram shown on the left.

### PULs identification and classification

PULs were broadly defined as genomic regions containing at least three genes coding for CAZymes, sulfatases, or transport proteins, following the criteria reported in the microbiome analysis of co-located green, brown, and red algae (Lu et al., 2023). These loci were assigned to four compositional categories: PULs comprising CAZymes and a *susCD* pair; CAZyme-rich gene clusters (CGCs) lacking transporter genes; PUL-like clusters containing CAZymes and a TonB-dependent receptor; and *susCD* loci without detectable CAZymes. Genes containing SusC/D-like proteins were predicted with HMMER v3.4 (Eddy, 2011) with an E-value cutoff of 1 × 10^−10^ against Pfam and TIGRFAM profiles. PFAM profiles used to identify sulfatase were PF00884, for SusD-like, PF07715, PF07980, PF12741, PF14322, PF12771, and TonB-dependent receptors were PF00593. The TIGRFAM profiles were TIGR04056, TIGR01352, TIGR01778, TIGR01779, TIGR01782, TIGR01783, TIGR01785, TIGR01786, TIGR02796, TIGR02797, TIGR02803, TIGR02804, TIGR02805, TIGR04057 (Table 2). CAZymes were identified with run_dbCAN^5^ (v5.2.1) with the dbCAN3 database (Zheng et al., 2023).

**Table 2:** Summary of Pfam and TIGRFAM profiles utilized in the identification of polysaccharide utilization loci (PULs), PUL-like clusters, and CAZyme gene clusters (CGCs), based on locus category.

Locus category	Defining features	Pfam profiles	TIGRFAM profiles
PULs	CAZymes + susCD pair	SusD-like: PF07715, PF07980, PF12741, PF14322, PF12771	SusC/D-like: TIGR04056, TIGR01352, TIGR01778, TIGR01779, TIGR01782, TIGR01783, TIGR01785, TIGR01786, TIGR02796, TIGR02797, TIGR02803, TIGR02804, TIGR02805, TIGR04057
PUL-like clusters	CAZymes + TonB-dependent receptor	TonB-dependent receptor: PF00593	N/A
CGCs	CAZymes	N/A	N/A

### Comparison with non-coenocytic algae

MAGs from *Ulva* sp. and *Saccharina* sp. of the orders Ulvales and Laminariales were used for comparative analysis of BGCs, PULs, and KEGG pathways, together with the non-redundant MAGs recovered from coenocytic algae. These MAGs were obtained from a reported microbiome analysis of co-located green, brown, and red algae (Lu et al., 2023). The MAGs from non-coenocytic algae were subjected to the same methodology as the MAGs from coenocytic algae for identification of BGCs, PULs, and KEGG pathways. Statistical comparisons of pathway completeness between coenocytic and non-coenocytic MAGs were performed in R v4.5.0 on the non-redundant MAG set, comprising 311 MAGs from coenocytic hosts and 201 from non-coenocytic hosts. A pathway was included in the comparison only if it was detected in at least half of the MAGs in each group, yielding 56 pathways for testing. Pathways falling below this threshold in either group were excluded. Each pathway was tested with a one-tailed Mann–Whitney *U* test using the normal approximation, with the alternative hypothesis that completeness is higher in coenocytic than in non-coenocytic MAGs. *p*-values were adjusted for multiple testing with the Benjamini–Hochberg method, and pathways with an adjusted *p*-value below 0.05 were considered significant. Effect sizes were calculated as rank-biserial correlations and classified as negligible below 0.1, small from 0.1 to below 0.3, medium from 0.3 to below 0.5, and large at 0.5 or above. Data processing and visualization used the R packages dplyr v1.2.0, tidyr v1.3.2 and ggplot2 v4.0.2.

## Results

### Coenocytic algae MAG quality and size distribution

The total number of MAGs recovered from the coenocytic WGS reads and from *V. bursata* LB2067 was 344 MAGs (Table 1, Supplementary Table S1, Figure 2H). A total of 303 MAGs were recovered from 11 algal datasets of the order Bryopsidales; 21 were near complete, 212 were HQ, 50 were GQ, and 20 were LQ. From the order Vaucheriales, a total of 41 MAGs were recovered; 32 were HQ, and 9 were GQ (Table 1, Supplementary Table S2) across the two *V. bursata* datasets. A total of 217 MAGs were from SR, 126 from LR, and one was a hybrid. MAGs from the order Bryopsidales and Vaucheriales showed similar quality in terms of N50, fragmentation, and genome size, with MAGs from Bryopsidales showing higher values for these metrics (Figures 2A–H). The recovery of a high number of HQ MAGs indicates that the proposed methodology can recover high-quality genomes from the WGS data of both *Bryopsidales* and *Vaucheriales.* This is further illustrated in the completion and contamination scatter plot (Figure 2G). In contrast to the quality of MAGs recovered, the genome size distribution between the two orders showed substantial variations, where MAGs from Bryopsidales algae have a genome size distribution of 1–10 Mbp, with fewer genomic elements of less than 1 Mbp (Supplementary Table S2). MAGs from Vaucheriales had a narrower genome size distribution, ranging from 3 to 9 Mbp, with a mean genome size of approximately 5 Mbp (Figure 2F).

**Figure 2:**
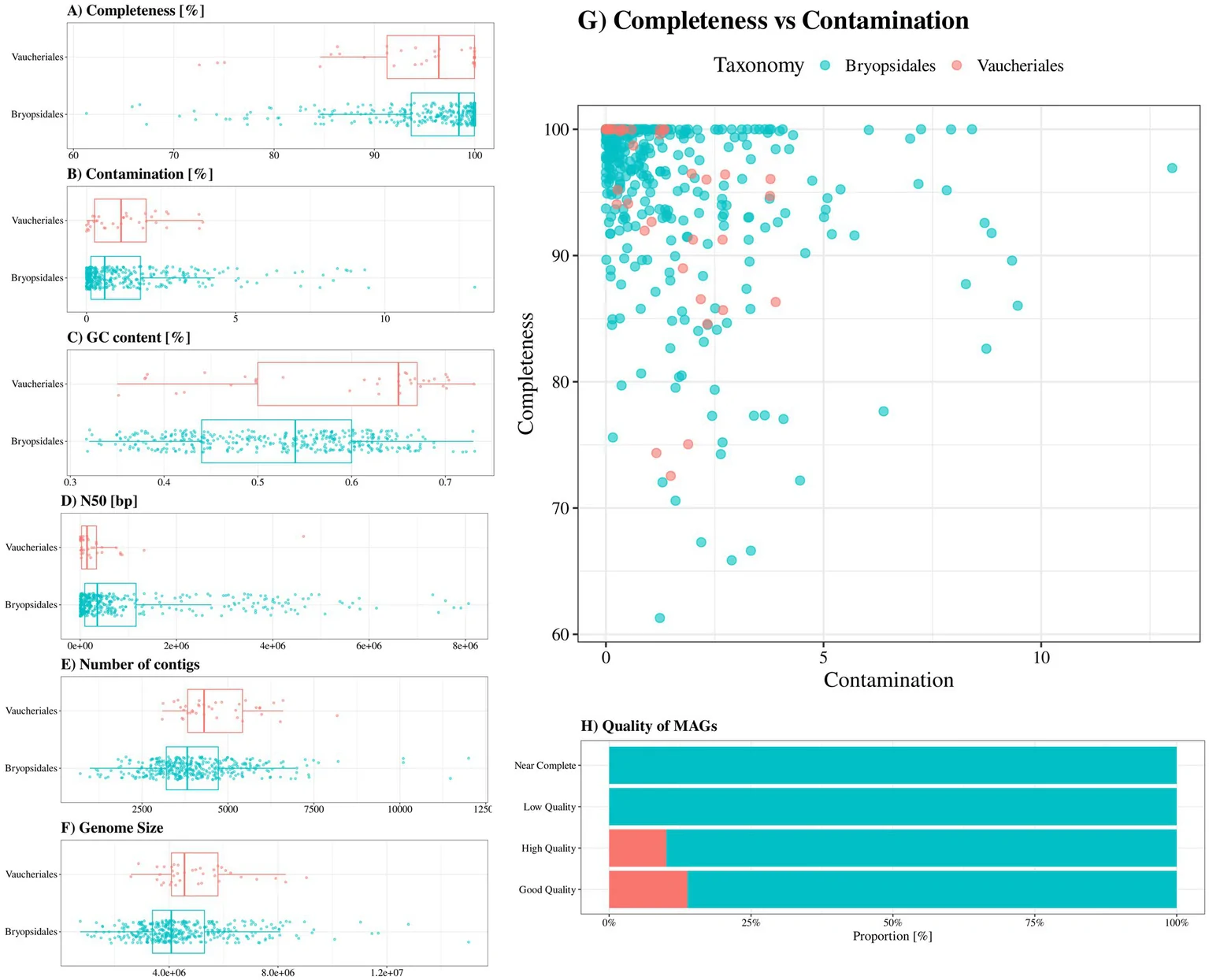
Quality, assembly, and genome characteristics of metagenome-assembled genomes (MAGs) recovered from coenocytic algal metagenomes. A total of 344 MAGs is shown, grouped by the taxonomic order of their algal hosts: 303 MAGs from 10 Bryopsidales species (teal) and 41 MAGs from 2 Vaucheriales species (orange). **(A–F)** Distributions of MAG quality and assembly metrics by host order. Boxes indicate the median and interquartile range (IQR), whiskers extend to 1.5 × IQR, and individual points represent MAGs. Metrics shown are **(A)** CheckM2-estimated completeness (%), **(B)** CheckM2-estimated contamination (%), **(C)** GC content, **(D)** assembly N50 (bp), **(E)** number of contigs, and **(F)** total genome size (bp). **(G)** Relationship between CheckM2-estimated completeness and contamination for individual MAGs, colored by host order. **(H)** Relative contribution of Bryopsidales- and Vaucheriales-associated MAGs within each quality tier. Quality tiers were defined using CheckM2 completeness and contamination estimates, with an additional single-contig criterion for near-complete MAGs: near complete (≥90% completeness, ≤5% contamination, single contig; *n* = 21, all Bryopsidales), high quality (HQ; >90% completeness and ≤5% contamination; *n* = 244: 212 Bryopsidales and 32 Vaucheriales), good quality (GQ; ≥50 to <90% completeness and ≤5% contamination; *n* = 59: 50 Bryopsidales and 9 Vaucheriales), and low quality (LQ; <50% completeness and ≤10% contamination; *n* = 20, all Bryopsidales). No error bars are shown.

### Taxonomic composition of the coenocytic algae

The taxonomic profiles of the recovered MAGs from the coenocytic orders Bryopsidales and Vaucheriales displayed broad similarities at the phylum level but diverged at finer taxonomic resolutions (Figures 3A,B). At the phylum level, both lineages were heavily dominated by Pseudomonadota and Bacteroidota. The extent of this dominance was comparable between the two orders; in the MAGs from Vaucheriales, these two phyla collectively comprise 75.6% of the microbiome, whereas in the MAGs from Bryopsidales, they account for 78.6%, with the latter showing a notable enrichment of Cyanobacteriota and Planctomycetota (Supplementary Tables S3, S4).

**Figure 3:**
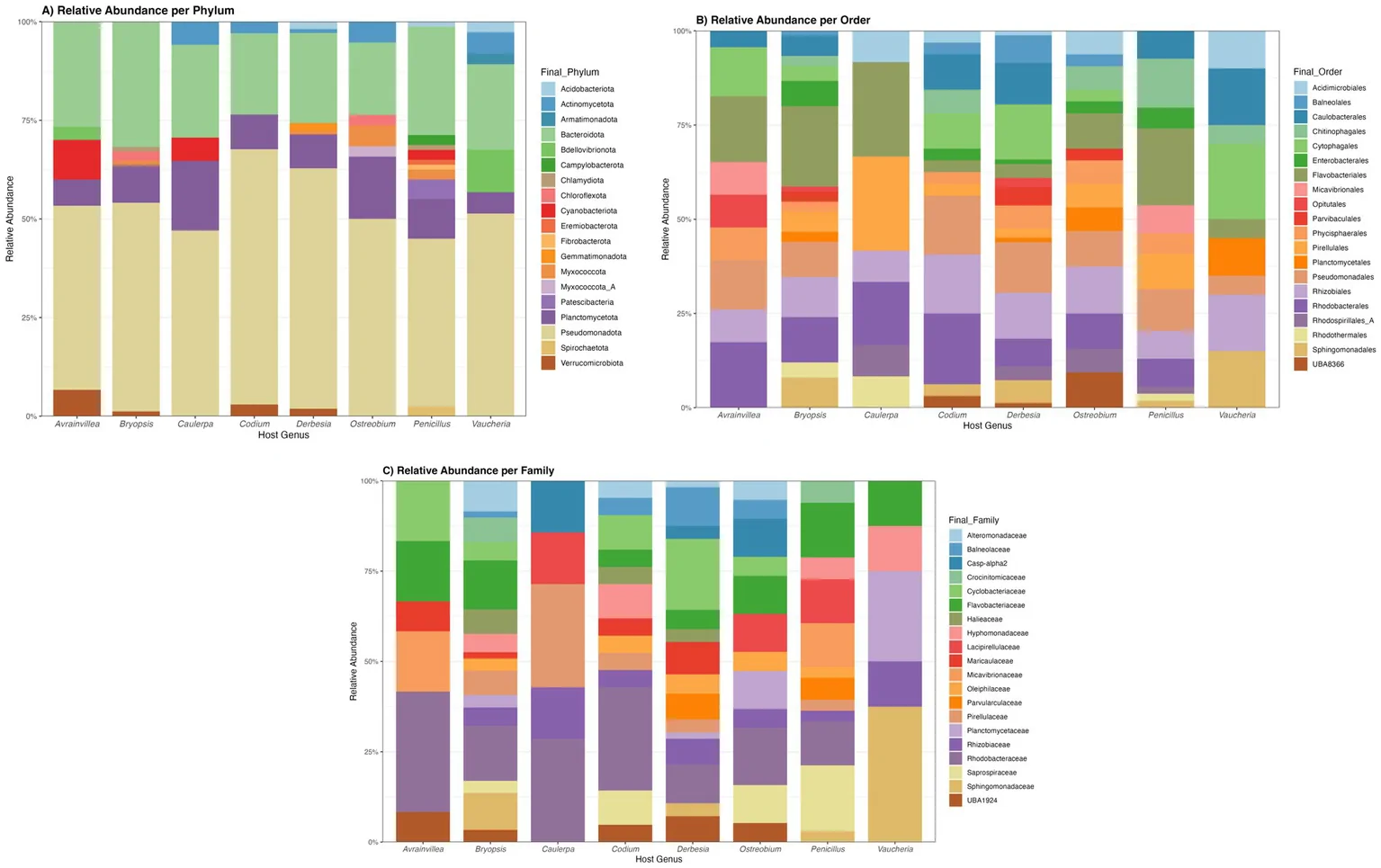
Taxonomic composition of bacterial metagenome-assembled genomes (MAGs) associated with coenocytic algae of the orders Bryopsidales and Vaucheriales. Taxonomic composition is based on 429 MAGs, comprising the 344 MAGs recovered in this study (Table 1) and 85 MAGs previously recovered from *Bryopsis* KO-2023 by Ochiai et al. (2025). MAGs were taxonomically classified using GTDB-Tk v2.6.1 with GTDB release 224. To support genus- and species-level assignments, 16S rRNA sequences were extracted using Barrnap v0.9 and classified against the SILVA 138.2 99% OTUs full-length sequence database. Stacked bars show the relative abundance of MAGs assigned to each taxon within each algal host genus, with each bar summing to 100%. MAGs from different host species were pooled by host genus. *Avrainvillea*, *Bryopsis*, *Caulerpa*, *Codium*, *Derbesia*, *Ostreobium*, and *Penicillus* belong to the Bryopsidales, whereas *Vaucheria* represents the Vaucheriales. **(A)** Relative abundance at the phylum level. **(B)** Relative abundance at the order level. **(C)** Relative abundance at the family level. For clarity, only the most abundant taxa at each taxonomic rank are displayed. Colors identify taxa within each panel according to the corresponding legend and should not be compared across taxonomic ranks.

At the order level (Figure 3B), the taxonomic divergence between Vaucheriales and Bryopsidales become more pronounced. MAGs from Vaucheriales were primarily distributed among Burkholderiales (12.2%), Sphingomonadales (9.8%), and Rhizobiales (7.3%), which together accounted for 29.3% of the recovered MAGs. In contrast, the Bryopsidales MAGs were co-dominated by Rhizobiales and Flavobacteriales (19.3% each), followed by Rhodobacterales (8.8%) and Pirellulales (4.4%). Flavobacteriales were also detected among Vaucheriales MAGs, although at a lower relative abundance. Taxonomic diversity was even more apparent at the family level (Figure 3C). Among Vaucheriales MAGs, Burkholderiaceae (12.2%) and Sphingomonadaceae (9.8%) were the most abundant families, followed by Bacteriovoracaceae (7.3%) and Rhizobiaceae (2.4%). In contrast, Bryopsidales MAGs showed greater representation of families associated with complex host interactions, including Rhodobacteraceae, Flavobacteriaceae, Pirellulaceae, and Oleiphilaceae.

### KEGG pathways from recovered MAGs of coenocytic orders

KEGG pathways from the coenocytic orders Bryopsidales and Vaucheriales were compared with those from the non-coenocytic hosts Ulva and Saccharina. All lineages encoded a conserved non-photosynthetic core of amino acid biosynthesis, glycolysis, and the TCA cycle (Supplementary Table S5). Removing pathways detected in all MAGs left only the metabolic capacities that differed among groups (Figure 1). The retained pathways cover polysaccharide degradation, sulfur and nitrogen cycling, nutrient acquisition, and secretion systems, with completeness ranging from absent to complete. Nitrogen fixation was complete in MAGs from *Derbesia* and *Bryopsis* sp. KO-2023 and partial in those from *Penicillus* and *Avrainvillea* (Supplementary Table S5). MAGs from the Vaucheriales carried complete nitrogen fixation pathways, alongside the cytochrome bd complex and polyhydroxybutyrate synthesis (Supplementary Table S5). Motility also differed. MAGs from non-coenocytic hosts carried complete flagellar assembly pathways, whereas those from the Bryopsidales retained only a partial flagellar and type III secretion signature (Supplementary Table S5).

A principal component analysis (PCA) of bacterial KEGG pathways revealed a broad functional similarity across MAGs from both non-coenocytic and coenocytic algae (Supplementary Figure S4). However, the MAGs from Vaucheriales displayed a distinct separation from the Bryopsidales. This divergence was most pronounced in the LB2067 dataset, which formed a cluster completely distinct from the main group, in contrast to MAGs from the other *V. bursata* datasets, which showed only moderate divergence and remained closer to the central functional array. Within the Bryopsidales, while most samples clustered tightly, specific *Caulerpa* species displayed notable variance, diverging from the major group arrays (Supplementary Figure S4).

A one-sided Mann–Whitney *U* test with Benjamini–Hochberg correction was used to identify pathways with higher completeness in coenocytic than in non-coenocytic MAGs. Testing was restricted to pathways detected in at least half of the MAGs in both groups. Of the 56 pathways that met this criterion, 46 were significantly more complete in coenocytic MAGs at an adjusted *p*-value below 0.05 (Supplementary Table S8). Thirty-eight of the 46 had a non-negligible effect size and are shown in Figure 4. Sec-SRP had the only large effect at *r* = 0.52, followed by flagellum, the F-type ATPase, the 3-hydroxypropionate bicycle, the TCA cycle, glycolysis, riboflavin biosynthesis, thiamin biosynthesis, valine, and isoleucine, all with medium effects. These pathways span protein secretion, motility, central carbon metabolism, and cofactor biosynthesis. The remaining eight significant pathways had negligible effects, and 10 pathways showed no significant difference.

**Figure 4:**
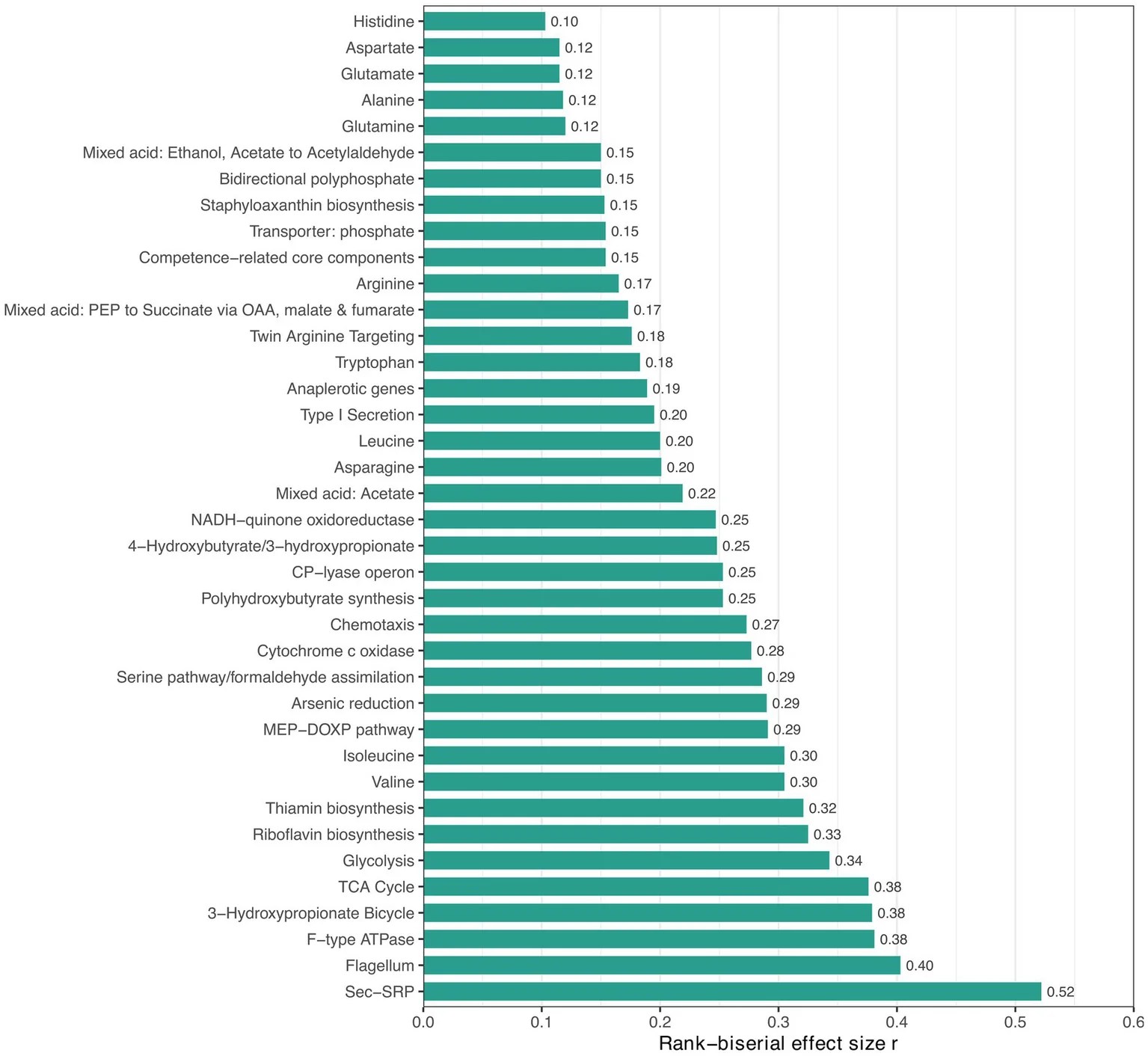
Metabolic pathways with significantly higher completeness in bacterial metagenome-assembled genomes (MAGs) from coenocytic algae. KEGG pathway completeness was compared using the non-redundant MAG set, comprising 311 MAGs from coenocytic hosts of the orders Bryopsidales and Vaucheriales and 201 MAGs from the non-coenocytic hosts *Ulva* sp. and *Saccharina* sp. Statistical testing was restricted to 56 pathways detected in at least 50% of MAGs in both host groups. For each pathway, completeness was compared using a one-tailed Mann–Whitney *U* test with the alternative hypothesis of higher completeness in MAGs from coenocytic hosts. *p-*values were adjusted for multiple comparisons using the Benjamini–Hochberg method. Of the 56 pathways tested, 46 were significantly enriched in coenocytic-associated MAGs (adjusted *p* < 0.05); the 38 pathways shown here additionally had a rank-biserial effect size (*r*) ≥ 0.10. Bars represent the rank-biserial correlation, with values shown at the end of each bar; larger positive values indicate a stronger shift toward greater pathway completeness in coenocytic-associated MAGs. Pathways are ordered by increasing effect size.

### BGC distribution from recovered MAGs from coenocytic order

A comparison of the distribution of identified biosynthetic gene clusters (BGCs) across the non-redundant MAGs showed a BGC density of 7.62 BGCs per MAG in Vaucheriales, 6.39 in Bryopsidales, 4.58 in Ulvales, and 4.23 in Laminariales (Figure 5A; Supplementary Table S6). The highest total number of BGCs was recovered from Bryopsidales (1,694 across 265 MAGs), followed by Ulvales (737 across 161 MAGs), Vaucheriales (221 across 29 MAGs), and Laminariales (127 across 30 MAGs). BGC density was 7.62 BGCs per MAG in Vaucheriales, 6.39 in Bryopsidales, 4.58 in Ulvales, and 4.23 in Laminariales (Figure 5A; Supplementary Table S6). Regarding BGC types, the majority were conserved between coenocytic and non-coenocytic algae, particularly terpenes, terpene precursors, and T1PKS (Figure 5B; Supplementary Table S6). The N-acetylglutaminylglutamine amide (NAGGN) BGC type was detected only in MAGs from coenocytic algae and was absent from non-coenocytic hosts. Its distribution among the coenocytic taxa was not uniform, as NAGGN was not detected in *Avrainvillea*, *Caulerpa*, and *Codium* species. The MAGs with NAGGN were assigned almost entirely to the Pseudomonadota and were distributed across several bacterial orders rather than being restricted to a single lineage.

**Figure 5:**
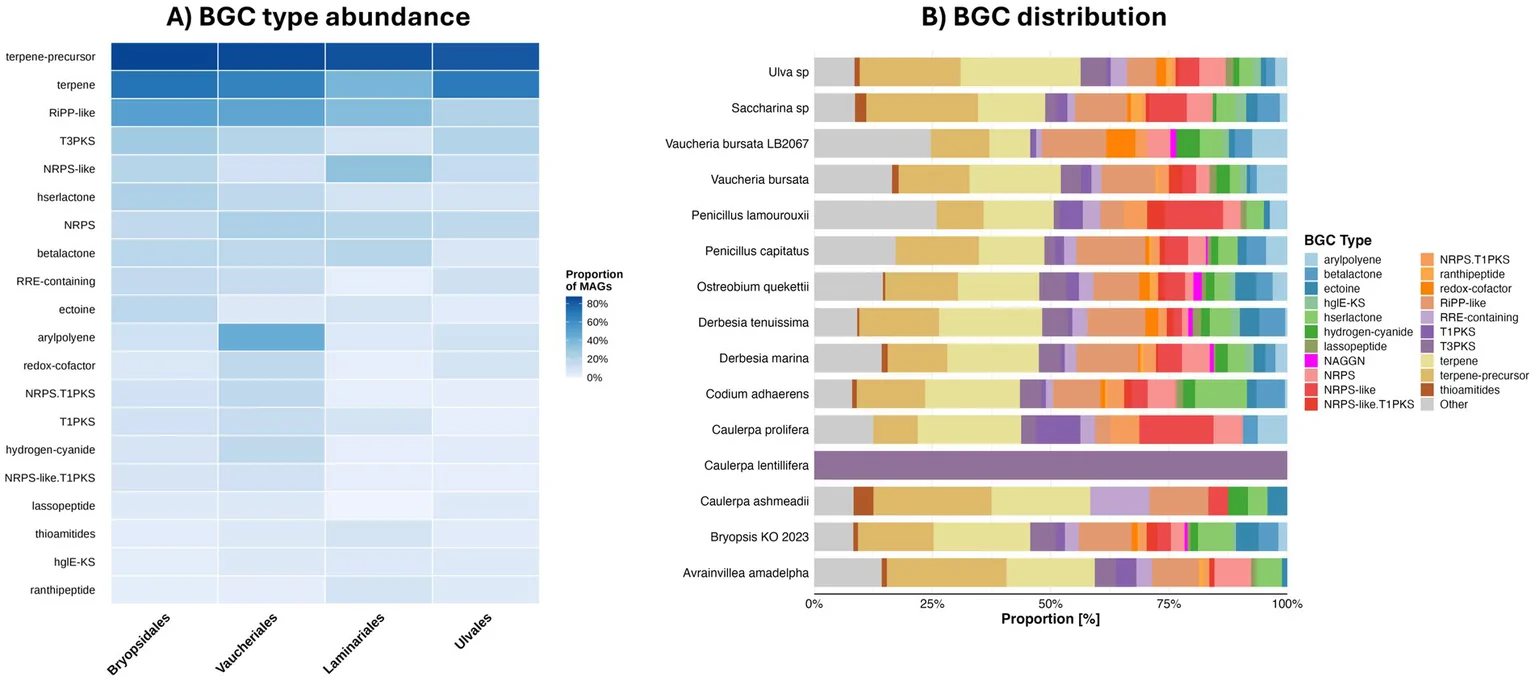
Distribution and composition of biosynthetic gene clusters (BGCs) across bacterial metagenome-assembled genomes (MAGs) from coenocytic and non-coenocytic algae. The non-redundant MAG set was used for this analysis, comprising 311 MAGs from coenocytic hosts and 201 MAGs from non-coenocytic hosts. BGCs were identified using antiSMASH v8.0.4 and classified into gene cluster families using BiG-SCAPE v2.0.0. **(A)** Prevalence of BGC types across algal host orders. Cells show the proportion of MAGs encoding at least one BGC of each type within the coenocytic orders Bryopsidales and Vaucheriales and the non-coenocytic orders Laminariales and Ulvales. Color intensity represents the percentage of MAGs carrying each BGC type, ranging from 0% (white) to 80% (dark blue). **(B)** Composition of BGCs by algal host species. Stacked bars show the relative frequency of the 20 most abundant BGC types among all BGCs detected in MAGs associated with each host species, with each bar summing to 100%. BGC types are distinguished by colors indicated in the legend, and types outside the 20 most abundant are grouped as “Other.” NAGGN, N-acetylglutaminylglutamine amide; NRPS, non-ribosomal peptide synthetase; PKS, polyketide synthase; T1PKS, type I polyketide synthase; T3PKS, type III polyketide synthase; RiPP, ribosomally synthesized and post-translationally modified peptide; RRE, RiPP recognition element; and hglE-KS, heterocyst glycolipid synthase-like ketosynthase.

The MAGs from the non-coenocytic algae, specifically *Ulva* sp., uniquely possessed T2PKS and PUFA clusters. In contrast, the MAGs from coenocytic algae were characterized by the exclusive presence of Butyrolactone and NAGGN clusters. Butyrolactone was specific to the MAGs from Bryopsidales, specifically *Bryopsis* KO-2023 (Supplementary Table S6). The NAGGN BGCs were more widely distributed among MAGs from coenocytic algae, with the highest prevalence in *Ostreobium* (33%) and *Derbesia* (32%), followed by *Bryopsis* (16%), and *Vaucheria* and *Penicillus* (8%). Despite the evolutionary distance between Vaucheriales and Bryopsidales, both shared the presence of NAGGN clusters. However, the MAGs from Vaucheriales were distinct, exclusively harboring clusters such as phosphonate-like, NRP-metallophore-NRPS-T1PKS, and various complex hybrid PKS/NRPS combinations.

### PULs distribution

The polysaccharide utilization potential of the bacterial community was assessed using the non-redundant MAGs to identify Polysaccharide Utilization Loci (PULs), PUL-like clusters, and CAZyme Gene Clusters (CGCs). Distinct distribution patterns were observed across all MAGs from coenocytic and non-coenocytic algae (Figure 6, Supplementary Table S7). The analysis revealed a marked predominance of PULs and PUL-like clusters compared to CGCs across the MAGs from algae of both coenocytic and non-coenocytic orders. As shown in Figure 6, the frequency of identified loci was consistently higher for PULs and PUL-like clusters than for CGCs. While CGCs were present across all groups, their overall abundance was notably lower than that of the PUL types.

**Figure 6:**
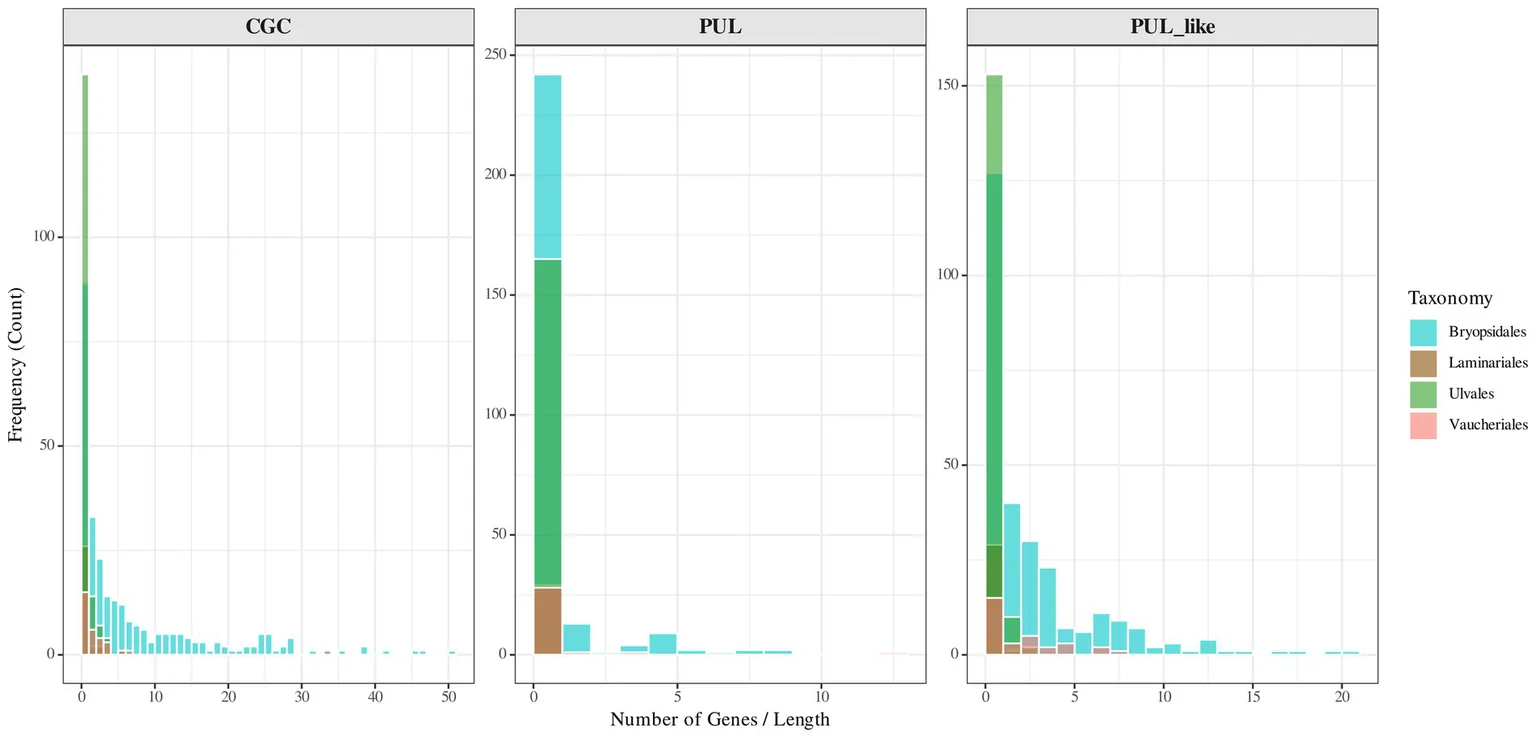
Size distributions of putative polysaccharide utilization loci and related carbohydrate-active gene clusters in bacterial metagenome-assembled genomes (MAGs) from coenocytic and non-coenocytic algae. The non-redundant MAG set was used for this analysis, comprising 311 MAGs from coenocytic hosts and 201 MAGs from non-coenocytic hosts. Putative loci were defined as genomic regions containing at least three genes encoding carbohydrate-active enzymes (CAZymes), sulfatases, or transport proteins, following Lu et al. (2023). CAZymes were identified using run_dbCAN v5.2.1 against the dbCAN3 database, whereas SusC-like proteins, SusD-like proteins, sulfatases, and TonB-dependent receptors were identified using HMMER against the Pfam and TIGRFAM profiles listed in Table 2. Loci are separated into three categories: *CGC*, CAZyme gene clusters containing CAZymes but no transporter genes; *PUL*, polysaccharide utilization loci containing CAZymes together with a SusC/SusD pair; and *PUL-like*, clusters containing CAZymes together with a TonB-dependent receptor. Histograms show the number of loci in each size class, with locus size expressed as the number of genes per locus and a bin width of [n] genes. Bars are stacked according to the taxonomic order of the algal host: Bryopsidales (blue) and Vaucheriales (pink), representing coenocytic hosts, and Laminariales (brown) and Ulvales (green), representing non-coenocytic hosts. Note that the y-axis scale differs among the three panels.

## Discussion

### Taxonomy classification and quality

The majority of MAGs recovered from the WGS reads of the coenocytic algae were classified as high- to good-quality MAGs, with a low level of contamination and high completeness (Figure 2G, Supplementary Table S2). This high yield of MAGs indicates the feasibility of recovering bacterial communities from host tissues, aligning with recent successes in both targeted coenocytic algal studies (Ochiai et al., 2025) and broad-scale macroalgal metagenomic surveys (Lu et al., 2023). The majority of MAGs recovered from SR was classified as good quality and high quality, in contrast to LR, for which MAGs were classified predominantly as high quality and near complete (Supplementary Figure S3). Both sequencing methods yielded LQ MAGs; the LR assemblies showed less fragmentation, with fewer contigs and higher N50 values than the SR assemblies (Supplementary Figure S3). This result is consistent with Orellana et al. (2023), who reported that short-read sequencing often recovers a higher total number of genomes due to greater sequencing depth, but long-read approaches yield a significantly higher proportion of high-quality MAGs. They reported that LR MAGs were composed of approximately 10 times fewer contigs and possessed N50 values more than 11 times higher than those of SR MAGs. Our results show that the workflow utilized for MAG recovery was able to yield HQ MAGs from both SR and LR WGS datasets.

The distribution of taxa in both coenocytic orders, Bryopsidales and Vaucheriales, showed strong similarities at the phylum level, characterized by a consistently high percentage of Pseudomonadota and Bacteroidota. In Bryopsidales, these two phyla constitute 78.6% of the recovered MAGs (53.1% Pseudomonadota and 25.5% Bacteroidota), while in the Vaucheriales, they account for 75.6% of the community (53.7% Pseudomonadota and 22.0% Bacteroidota) (Figure 3A). These findings align with previous research on Bryopsidales in which the community of *Caulerpa lentillifera* was dominated by Pseudomonadota (syn. Proteobacteria) at 52.1% and Bacteroidota (syn. Bacteroidetes) at 13.5% (Liang et al., 2019). The dominance of Pseudomonadota observed in the *V. bursata* MAGs aligns with previous 16S rRNA sequencing of *V. litorea*, which reported an 88.4% relative abundance of this phylum. However, *V. bursata* exhibited a notably higher proportion of Bacteroidota (22.0%) compared to the 7.3% observed in *V. litorea* (Devine et al., 2012). This could indicate a difference in species recruitment, though this variance may also be influenced by the differences between WGS binning and 16S rRNA sequencing. The co-dominance of Pseudomonadota and Bacteroidota is not unique to coenocytic algae but has been reported across diverse algal lineages, such as in the red alga *Pyropia haitanensis*, comprising approximately 65% of the sequenced microbiome (Wang et al., 2022). Furthermore, a major comparative study across *Ulva* sp., *Grateloupia* sp., *Gelidium* sp., and *Saccharina* sp. found that most core genera belonged to Pseudomonadota and Bacteroidota, and that these core genera dominated the algal surface, comprising 51.1% of the total bacterial community compared to only 5.7% in the surrounding seawater and 1.5% in the sediment (Lu et al., 2023). The phylum distribution of MAGs between coenocytic algae is consistent with other non-coenocytic marine algae with a dominance of Pseudomonadota and Bacteroidota, even in the case of *V. bursata* LB2067, which resides in freshwater.

In contrast to the phylum level, at the order level, MAGs from Bryopsidales show a specific dominance by Rhizobiales and Flavobacteriales, both at 19.3% across all algae, followed by Rhodobacterales at 8.8% and a substantial representation of Pseudomonadales (Figure 3B). Conversely, MAGs from Vaucheriales show a distribution of Burkholderiales at 12.2%, Sphingomonadales at 9.8%, and Rhizobiales at 7.3%, cumulatively 29.3% (Figure 3B). Rhodobacterales has likewise been reported as a dominant order in Bryopsidales, accounting for 22.0% of the reads in *Caulerpa lentillifera* (Liang et al., 2019). Taxonomic profiling of the *V. bursata* MAGs revealed high abundances of Rhizobiales and Burkholderiales (Figure 3B, Supplementary Table S4), alongside a lower abundance of Sphingomonadales (Figure 3B), compared to the known composition of *V. litorea* (Devine et al., 2012). Furthermore, while Rhodobacterales was reported in *V. litorea*, this order was absent from the *V. bursata* MAGs. These distinctions could indicate a divergence between the bacterial communities associated with these two *Vaucheria* species.

At the family level, there is more diversity among all MAGs from coenocytic algae for both Bryopsidales and Vaucheriales (Figure 3C). Within Bryopsidales, Flavobacteriales are present in *Bryopsis* sp. KO-2023 species and the genus *Penicillus*. In contrast, the family Flavobacteriaceae from the order Flavobacteriales is present across both Bryopsidales and Vaucheriales. In 2011, a bacterial endophyte from the order Flavobacteriales was reported to be present in *Bryopsis*. The widespread presence of the Flavobacteriaceae family across both Bryopsidales and Vaucheriales (Supplementary Table S4) could indicate that a member of this order could be a potential endophyte or symbiont associated with coenocytic algae (Hollants et al., 2011).

Together, all these findings highlight complex biological interactions; notably, the overall taxonomic distribution observed in these MAGs remains inextricably linked to the abiotic environmental conditions, such as salinity and pH, surrounding the algae prior to sequencing. Consequently, the taxonomic profiles generated here require further validation using complementary methodologies.

### Metabolic KEGG pathways distribution

An evaluation of KEGG metabolic pathways across the MAGs recovered from coenocytic and non-coenocytic algae suggests that core bacterial metabolism remains highly conserved. Pathways for glycine, cysteine, and lysine biosynthesis show high completeness across all MAGs, as do glycolysis and the TCA cycle (Supplementary Table S5). Against this conserved background, a non-parametric Mann–Whitney *U* test with Benjamini–Hochberg correction was applied to pathways detected in at least half of the MAGs in both groups. Of the 56 pathways that met this criterion, 46 were significantly more complete in coenocytic-associated MAGs. Thirty-eight of these had non-negligible effect sizes (Supplementary Table S8). The Sec-SRP protein secretion system showed the strongest signal. This may indicate enhanced secretory capacity among bacteria associated with coenocytic algae. Such capacity could reflect a closer interaction with the host environment. Motility traits were also enriched, including flagellar assembly and chemotaxis. Both have been associated with host colonization. Bidirectional polyphosphate metabolism and riboflavin biosynthesis were likewise more complete in coenocytic-associated MAGs, with effect sizes of 0.15 and 0.33 (Figure 4). The enrichment of the polyP pathway is notable. It has often been described as an indicator of environmental durability typically associated with a free-living lifestyle (Wang et al., 2018). Its enrichment here could imply that the microbiomes of coenocytic algae retain a broad survival capacity despite their host association.

Polyhydroxybutyrate (PHB) synthesis was significantly enriched in coenocytic-associated MAGs compared to non-coenocytic controls (Figure 4). Within the coenocytic group, all MAGs from Vaucheriales showed a consistent completeness of 83% for the PHB synthesis pathway. Conversely, 46.9% of MAGs from non-coenocytic algae showed low completeness in this pathway, with values of 15.4 and 6.2% for Ulvales and Laminariales, respectively (Supplementary Table S5). Various studies have reported that PHB synthesis serves as a critical survival mechanism, providing a carbon reserve through the accumulation of PHB polymer (Verlinden et al., 2007; Timmis, 2010; Müller-Santos et al., 2021). The characteristics of PHB synthesis have been demonstrated in several bacterial species, such as *Agromyces indicus*. In 2021, PHB synthesis was optimized for *Agromyces indicus* using response surface methodology (RSM). Researchers concluded that the subsequent decrease in PHB was a direct response to carbon and nitrogen limitation and represented a survival mechanism under the conditions of the RSM analysis (Adnan et al., 2022). The higher prevalence of MAGs with pathway completeness for PHB synthesis (Figure 4) suggests that the Vaucheriales bacterial community exhibits mechanisms for growth in environments with a high density of organisms, potentially in algal mats. Throughout all MAGs from the coenocytic and non-coenocytic algae, notably only one MAG from *V. bursata* LB2067 showed a complete pathway with Biofilm PGA. The MAG was taxonomically predicted by GTDB-Tk to be *Achromobacter aegrifaciens*. It was not recovered from *V. bursata* MAGs, in contrast to *V. bursata* LB2067 MAGs. That metagenome was derived from a field-collected sample, whereas *V. bursata* LB2067 is a laboratory culture. Given this, the bacterium could have been acquired during long-term maintenance of *V. bursata* LB2067 in culture. Reagent and kit-associated contamination is a further possibility and cannot be excluded. The bacterium may instead occur naturally at low frequency in *V. bursata*. To investigate this further, additional isolates and negative controls would be needed to distinguish among these explanations.

### BGC and PUL distribution

BGC density was highest in Vaucheriales (7.62 BGCs per MAG) and Bryopsidales (6.39 per MAG), compared with Ulvales (4.58 per MAG) and Laminariales (4.23 per MAG). MAGs from coenocytic hosts therefore encoded approximately 1.4 times as many BGCs per genome as those from non-coenocytic hosts (6.51 versus 4.52 BGCs per MAG), with a similar difference in the number of distinct BGC types per MAG (5.18 versus 3.55). The larger absolute number of BGCs recovered from Bryopsidales (1,694) reflects the greater number of hosts and MAGs available for that order (11 hosts) rather than a higher biosynthetic density per genome. Vaucheriales MAGs showed the highest BGC density of any order, at 7.62 BGCs and 6.07 BGC types per MAG, despite being recovered from only two host datasets. MAGs from the analyzed algae of the Bryopsidales and Vaucheriales contain the NAGGN BGC type, in contrast to MAGs from their non-coenocytic counterparts (Figure 5B). NAGGN was not detected in *Avrainvillea*, *Caulerpa*, or *Codium* species. The MAGs possessing NAGGN BGCs were assigned to the Pseudomonadota, spanning several orders including the Rhizobiales, Pseudomonadales, Caulobacterales, Nevskiales, Rhodospirillales, and Acidimicrobiales. The NAGGN BGCs are associated with osmoprotection mechanisms via the integration of compatible solutes, enabling bacteria to regulate internal osmotic pressure. Together with the KEGG pathway analysis of the MAGs, this could indicate that bacterial communities in coenocytic algae are capable of surviving in complex environments, from permanently saline oceans to soil and the interior of plant or animal hosts (Sagot et al., 2010). Regarding carbohydrate-active gene systems, MAGs from coenocytic algae showed a higher abundance of PULs and PUL-like systems than CGCs (Figure 6). While canonical PULs are characteristic of Bacteroidota, PUL-like clusters are more broadly distributed across bacterial lineages, including Pseudomonadota (Martens et al., 2011). The predominance of PUL-like systems could therefore be consistent with the high relative abundance of Pseudomonadota in these communities (Figure 3A). This may suggest a community-wide reliance on complex-carbohydrate-degradation machinery in the bacterial community of coenocytic algae. Notably, MAGs tend to be more fragmented. Because PULs and PUL-like systems are defined over co-located arrays of genes, assembly fragmentation can split these loci across contigs, so that they may be recovered as shorter clusters than those actually present. Similar limitations of MAG-based gene-cluster prediction have been noted previously (Lu et al., 2023).

A comprehensive analysis of recovered MAGs from SR and LR of coenocytic algae from the Vaucheriales and Bryopsidales orders showed a high dominance of Pseudomonadota at the phylum level. Conversely, no consistent pattern was evident in the order- or family-level taxonomy predicted by GTDB-Tk. Moreover, this lack of pattern in taxonomic distribution could also be attributable to environmental factors associated with the algal sequencing procedure. KEGG pathways in MAGs from Bryopsidales and Vaucheriales showed high completeness and were significantly enriched in coenocytic-associated MAGs, including protein secretion (Sec-SRP), motility (Flagellar assembly, Chemotaxis), and nutrient-provisioning pathways (thiamin biosynthesis, MEP-DOXP, phosphate transporters), suggesting that the coenocytic morphology could potentially favor bacteria with enhanced host-interaction and biosynthetic capacities. In MAGs from Vaucheriales, the high prevalence of PHB synthesis may reflect a survival mechanism that provides a carbon reserve in low nutrient environments. In addition, the high number of MAGs exhibiting 83% completeness of the PHB biosynthesis pathway suggests that *Vaucheria* species may represent a potential source of bacteria with PHB-producing capabilities, given the importance of PHB in bioeconomic applications (Verlinden et al., 2007). NAGGN BGCs were detected only in MAGs from algae of the coenocytic order. NAGGN has been associated with osmoprotection. The high completeness of specific KEGG pathways and NAGGN BGCs within MAGs of the coenocytic algae suggests that a bacterial community is equipped with various survival mechanisms adapted to diverse and challenging environments. Given the observed adaptation of survival mechanisms, we hypothesize that these patterns may, in part, reflect the cosmopolitan distribution of the diverse coenocytic algal hosts from which the MAGs were recovered. To maximize representation of publicly available genomic data from coenocytic algae, MAGs derived from both short- and long-read sequencing datasets were included in our analyses. However, because short-read assemblies are generally more fragmented, differences in assembly contiguity between sequencing approaches may affect estimates of KEGG pathway completeness, biosynthetic gene cluster (BGC) abundance, and polysaccharide utilization locus (PUL) detection. Accordingly, the associations identified here between coenocytic morphology and the functional potential of their associated bacterial communities should be interpreted as hypotheses that warrant further investigation and experimental validation.

This study represents an initial step toward elucidating the complexity of the microbiome associated with coenocytic algae from the orders Bryopsidales and Vaucheriales, as well as exploring potential associations with host coenocytic morphology.

## Data Availability

The dataset generated in this study was deposited in the NCBI SRA database with the following accession SRX30956066. The MAG sequences, their structural annotations, and the scripts used for MAG recovery are available on Figshare: https://doi.org/10.6084/m9.figshare.31953375.

## Footnotes

1
https://github.com/wwood/galah

2
https://github.com/tseemann/barrnap

3
https://github.com/iliapopov17/KEGGaNOG

4
https://github.com/medema-group/BiG-SCAPE

5
https://github.com/bcb-unl/run_dbcan
