# Supplementary material for: Genomic insights into bacterial communities of coenocytic algae using metagenome-assembled genomes
Source: Front Microbiol. 2026 Oct 6;17:1851259. doi: 10.3389/fmicb.2026.1851259 (PMC13680199; doi:10.3389/fmicb.2026.1851259)
Supplement: Supplementary file 1 [file Supplementary_file_1.DOCX]

***Supplementary Material***

# 1 Supplementary Data

**Taxonomy prediction by GTDB-Tk**

The taxonomic profiles of the coenocytic orders Bryopsidales and Vaucheriales, determined by GTDB-Tk, display broad similarities at the phylum level but diverge at finer taxonomic resolutions. At the phylum level, both lineages are heavily dominated by Pseudomonadota and Bacteroidota, and the extent of this dominance is comparable. These two phyla collectively comprise 78.6% of the Bryopsidales assemblage, with Pseudomonadota at 53.1% and Bacteroidota at 25.5%. In the Vaucheriales, they comprise 75.6%, with Pseudomonadota at 53.7% and Bacteroidota at 22.0%. The remaining fraction differs between the two orders. Vaucheriales retain Bdellovibrionota at 9.8%, Actinomycetota and Planctomycetota each at 4.9%, and minor traces of Acidobacteriota and Armatimonadota each at 2.4%. Bryopsidales instead retain Planctomycetota at 10.1%, followed by Myxococcota, Cyanobacteriota and Verrucomicrobiota each at 1.6% and Actinomycetota at 1.3%, with a further 5.4% distributed among phyla below this threshold.

At the order level, the divergence becomes more pronounced. The MAGs from Vaucheriales are led by Burkholderiales at 12.2%, followed by Sphingomonadales and Cytophagales each at 9.8%. A secondary tier comprises Rhizobiales, Caulobacterales, and the predatory order Bacteriovoracales, each at 7.3%. In contrast, the Bryopsidales assemblage is led by Flavobacteriales at 10.6% and Pseudomonadales at 9.0%, followed by Rhizobiales and Rhodobacterales each at 8.8%, Cytophagales at 5.7% and Pirellulales at 4.4%. The abundance of Flavobacteriales in Bryopsidales is consistent with previous culture-dependent studies identifying Flavobacteriaceae as core facultative endophytes in siphonous algae (Hollants et al., 2011). Sphingomonadales is markedly less abundant in Bryopsidales at 3.4%, and Burkholderiales was not detected in any Bryopsidales MAGs despite being the leading order in the Vaucheriales.

At the family level, the biological distinction is sharpest. The Vaucheriales are dominated by Burkholderiaceae at 12.2% and Sphingomonadaceae at 9.8%, each matching its parent order. The assemblage also includes Bacteriovoracaceae at 7.3%, Caulobacteraceae and Spirosomataceae each at 4.9%, and Rhizobiaceae at 2.4%, indicating that most Rhizobiales MAGs fall outside that family. Conversely, the Bryopsidales display Rhodobacteraceae at 8.8%, Flavobacteriaceae at 5.4%, Cyclobacteriaceae at 4.9% and the Planctomycetota-derived Pirellulaceae at 2.6%. All Rhodobacterales MAGs resolve to Rhodobacteraceae, whereas only half of the Flavobacteriales MAGs resolve to Flavobacteriaceae.

**Distribution of Metabolic KEGG Pathways Across Coenocytic and Non-Coenocytic Orders**

Metabolic reconstruction across the coenocytic orders Bryopsidales and Vaucheriales, alongside the non-coenocytic algal microbiomes of Ulva and Saccharina, revealed universally conserved pathways in all lineages, including amino acid biosynthesis (glycine, lysine, and serine), glycolysis, and the TCA cycle. However, distinct patterns of nitrogen metabolism differentiated the algal hosts. In the Bryopsidales, *Derbesia* and *Bryopsis* KO-2023 *sp.* encoded complete nitrogen fixation pathways, contrasting with the partial completeness ranging from 0.3 to 0.6 observed in *Penicillus* and *Avrainvillea*, and the genomically reduced profiles of the MAGs from *Ostreobium*. In the Vaucheriales, nitrogen fixation appeared strictly context-dependent, with *Vaucheria bursata* LB2067 exhibiting complete nitrogenase modules co-occurring with Cytochrome bd ubiquinol oxidase and Polyhydroxybutyrate synthesis.

Comparison of the community structure of MAGs recovered from coenocytic algae using PCA (Figure S4) revealed a pattern consistent with shared KEGG pathways. The majority of MAGs from coenocytic and non-coenocytic algae shared similar functional profiles, with the exception of both *Caulerpa lentillifera* and *Caulerpa prolifera*. Conversely, the Vaucheria bursata LB2067 community profile, located at the upper end of the PCA clustering, showed a distinct separation from the other coenocytic communities, including its wild counterpart, Vaucheria bursata.

**BGC Distribution Across Coenocytic and Non-Coenocytic Orders**

We evaluated the distribution of Biosynthetic Gene Clusters (BGCs) between the MAGs from the algae of the coenocytic orders Bryopsidales and Vaucheriales and non-coenocytic orders Ulvales and Laminariales to assess any specific traits. Notably, despite the lower representation of algal hosts in Vaucheriales and Ulvales (two and one hosts, respectively), their bacterial communities exhibited a remarkably high density of BGCs relative to the number of algae compared to those in Bryopsidales. When comparing the BGC composition across lineages, the majority of the bacterial community showed high similarity in conserved clusters, specifically terpenes, terpene precursors, and type I polyketide synthases (T1PKS), which were present in both coenocytic and non-coenocytic lineages (Figure 4B).

In contrast, both coenocytic orders were characterized by the specific presence of Butyrolactone and N-acetylglutaminylglutamine amide (NAGNN) gene clusters. Butyrolactone was exclusive to the bacterial community associated with Bryopsis KO-2023 sp. NAGNN clusters, while low in total count (n = 12), were more broadly distributed among the coenocytic algae. The distribution of NAGNN was highest in *Ostreobium* (33%) and *Derbesia* (32%), followed by the *Bryopsis* genus (16%), and *Vaucheria* and *Penicillus* (8% each).

Finally, MAGs from coenocytic algae belonging to both Vaucheriales and Bryopsidales shared the NAGNN cluster. However, the MAGs from the Vaucheriales differentiated themselves through specific complex gene clusters not found in Bryopsidales. These included phosphonate-like clusters and complex hybrid architectures such as NRP-metallophore-NRPS-T1PKS and T3PKS-terpene hybrids.

# 2 Supplementary Figures and Tables

## 2.1 Supplementary Figures

**Figure S1. Workflow for MAG recovery from short-read and long-read sequencing data.** Three strategies were applied according to data availability for each algal host. In the short-read pipeline, Illumina reads were processed with the metaWRAP Read_QC, Assembly, Binning, Bin_Refinement, and Reassembly_Bin modules, and bins larger than 10 Mbp were removed to exclude potential eukaryotic contamination and assembly artifacts. In the long-read pipeline, PacBio or Nanopore reads were assembled with Flye using the --meta option, binned in parallel with MaxBin2 and MetaBAT2, consolidated with DAS Tool, and filtered at the same 10 Mbp threshold. Long-read datasets without matching short reads were reported as final long-read-only MAGs. Where both data types were available, the resulting bin sets entered the refinement pipeline, in which FastANI identified pairs of long-read and short-read bins sharing at least 95% average nucleotide identity. For each pair, the long reads were mapped to the long-read bin with minimap2 and the short reads to the short-read bin with Bowtie2. Mapped reads were extracted with SAMtools and reassembled with Unicycler. Each reassembled bin was then evaluated with CheckM2 and compared with its original pair based on completeness, contamination, genome size, N50, and contig count, and the highest-quality version was retained for the final bin set. Solid arrows indicate the flow of data between steps, dashed boxes indicate intermediate outputs, and diamonds indicate decision points.

**Figure S2. Workflow for functional annotation, taxonomic prediction, and gene cluster discovery from the recovered MAGs.** The set of non-redundant MAGs was used as input for all analyses shown. Structural annotation was performed with Bakta using the --metagenome option, and the predicted genes were then passed to four parallel branches. CAZymes and polysaccharide utilization loci were predicted with run_dbCAN against the dbCAN3 database. Taxonomic classification was performed with GTDB-Tk, and 16S rRNA sequences were classified against the SILVA 138.2 99% OTUs full-length sequences database for validation at the genus and species levels. Functional annotation was performed with eggNOG-mapper, and its output was summarized to KEGG pathway completeness with KEGGaNOG. Biosynthetic gene clusters were predicted with antiSMASH, and the resulting clusters were classified into families with BiG-SCAPE. Arrows indicate the flow of data between steps.

**Figure S3. Quality metrics of MAGs recovered from long-read and short-read whole-genome sequencing data of coenocytic algae.** All MAGs recovered from hosts of the orders Bryopsidales and Vaucheriales are shown, grouped by sequencing strategy: 126 MAGs from long reads (LR; blue) and 217 MAGs from short reads (SR; red). In **(A)** and **(B)**, boxes show the median and interquartile range, whiskers extend to 1.5 × IQR, and each point is one MAG. **(A)** Distribution of contig N50, in bp. **(B)** Total number of contigs per MAG. **(C)** Distribution of MAGs across quality tiers, with each point representing one MAG and points spread horizontally within a tier to reduce overplotting. Quality tiers are defined in the Materials and Methods.

**Figure S4. Principal component analysis of KEGG pathway completeness profiles from MAGs of coenocytic and non-coenocytic algae.** The set of non-redundant MAGs was used for this analysis. Each point represents one algal host and gives the community consensus profile, obtained by aggregating the KEGG pathway completeness values of all MAGs recovered from that host. Points are colored by the taxonomic order of the host: Bryopsidales in blue, Vaucheriales in pink, Ulvales in green, and Laminariales in brown. Axes give the first two principal components, which explain 11.3% and 5.8% of the total variance. The shaded ellipse is a 95% confidence ellipse. Species names are given beside each point.

## 2.2 Supplementary Tables

**Table S1**. Sequencing datasets used for MAG recovery, including NCBI accession numbers and sequencing platforms for each algal host.

| **Organism** | **Order Taxonomy** | **Newly Sequenced** | **Previously sequenced** | **BioProject** | **BioSample** | **SRA** | **Platform** |
| --- | --- | --- | --- | --- | --- | --- | --- |
| *Vaucheria bursata* LB2067 | Vaucheriales | Y | N | [PRJNA1020787](https://www.ncbi.nlm.nih.gov/bioproject/PRJNA1020787) | [SAMN52960556](https://www.ncbi.nlm.nih.gov/biosample/SAMN52960556) | [SRX30956066](https://www.ncbi.nlm.nih.gov/sra/SRX30956066%5baccn%5d) | Illumina |
| *Vaucheria bursata* | Vaucheriales | N | Y | [PRJNA894593](https://www.ncbi.nlm.nih.gov/bioproject/PRJNA894593) | [SAMN35990819](https://www.ncbi.nlm.nih.gov/biosample/SAMN35990819) | [SRX20791602](https://www.ncbi.nlm.nih.gov/sra/SRX20791602%5baccn%5d) | Illumina |
| *Ostreobium quekettii* | Bryopsidales | N | Y | [PRJEB39309](https://www.ncbi.nlm.nih.gov/bioproject/PRJEB39309) | [SAMEA7428556](https://www.ncbi.nlm.nih.gov/biosample/SAMEA7428556) | [ERX4604558](https://www.ncbi.nlm.nih.gov/sra/ERX4604558%5baccn%5d) | Nanopore |
|  |  | N | Y | [PRJEB39309](https://www.ncbi.nlm.nih.gov/bioproject/PRJEB39309) | [SAMEA7051192](https://www.ncbi.nlm.nih.gov/biosample/SAMEA7051192) | [ERX4278978](https://www.ncbi.nlm.nih.gov/sra/ERX4278978%5baccn%5d) | Illumina |
| *Derbesia tenuissima* | Bryopsidales | N | Y | [PRJNA929663](https://www.ncbi.nlm.nih.gov/biosample/SAMD00079363) | [SAMN32957814](https://www.ncbi.nlm.nih.gov/biosample/SAMD00079363) | [SRX19215829](https://www.ncbi.nlm.nih.gov/sra/SRX19215829%5baccn%5d) | Illumina |
| *Derbesia marina* | Bryopsidales | N | Y | [PRJNA929663](https://www.ncbi.nlm.nih.gov/biosample/SAMD00079363) | [SAMN32957297](https://www.ncbi.nlm.nih.gov/biosample/SAMD00079363) | [SRX19217704](https://www.ncbi.nlm.nih.gov/sra/SRX19217704%5baccn%5d) | Illumina |
| *Codium adhaerens* | Bryopsidales | N | Y | [PRJNA929663](https://www.ncbi.nlm.nih.gov/biosample/SAMD00079363) | [SAMN32957322](https://www.ncbi.nlm.nih.gov/biosample/SAMN32957322) | [SRX19215809](https://www.ncbi.nlm.nih.gov/sra/SRX19215809%5baccn%5d) | Illumina |
| *Caulerpa lentillifera* | Bryopsidales | N | Y | [PRJDB5734](https://www.ncbi.nlm.nih.gov/biosample/SAMD00079363) | [SAMD00079363](https://www.ncbi.nlm.nih.gov/biosample/SAMD00079363) | [DRX143537](https://www.ncbi.nlm.nih.gov/sra/DRX143537%5baccn%5d) | PacBio |
|  |  | N | Y | [PRJDB5734](https://www.ncbi.nlm.nih.gov/biosample/SAMD00079363) | [SAMD00079363](https://www.ncbi.nlm.nih.gov/biosample/SAMD00079363) | [DRX143535](https://www.ncbi.nlm.nih.gov/sra/DRX143535%5baccn%5d) | Illumina |
|  |  | N | Y | [PRJDB5734](https://www.ncbi.nlm.nih.gov/biosample/SAMD00079363) | SAMD00079363 | [DRX143534](https://www.ncbi.nlm.nih.gov/sra/DRX143534%5baccn%5d) | Illumina |
|  |  | N | Y | [PRJDB5734](https://www.ncbi.nlm.nih.gov/biosample/SAMD00079363) | SAMD00079363 | [DRX143533](https://www.ncbi.nlm.nih.gov/sra/DRX143533%5baccn%5d) | Illumina |
| *Caulerpa ashmeadii* | Bryopsidales | N | Y | [PRJNA515488](https://www.ncbi.nlm.nih.gov/biosample/SAMD00079363) | [SAMN10768126](https://www.ncbi.nlm.nih.gov/biosample/SAMN10768126) | [SRX6913370](https://www.ncbi.nlm.nih.gov/sra/SRX6913370%5baccn%5d) | Illumina |
|  |  | N | Y | [PRJNA515488](https://www.ncbi.nlm.nih.gov/biosample/SAMD00079363) | [SAMN10768126](https://www.ncbi.nlm.nih.gov/biosample/SAMN10768126) | [SRX6913371](https://www.ncbi.nlm.nih.gov/sra/SRX6913371%5baccn%5d) | Illumina |
|  |  | N | Y | [PRJNA515488](https://www.ncbi.nlm.nih.gov/biosample/SAMD00079363) | [SAMN10768126](https://www.ncbi.nlm.nih.gov/biosample/SAMN10768126) | [SRX6913372](https://www.ncbi.nlm.nih.gov/sra/SRX6913372%5baccn%5d) | Illumina |
| *Avrainvillea amadelpha* | Bryopsidales | N | Y | [PRJNA929663](https://www.ncbi.nlm.nih.gov/biosample/SAMD00079363) | [SAMN32966959](https://www.ncbi.nlm.nih.gov/biosample/SAMN32966959) | [SRX19230453](https://www.ncbi.nlm.nih.gov/sra/SRX19230453%5baccn%5d) | Illumina |
| *Penicillus lamourouxii* | Bryopsidales | N | Y | [PRJEB88235](https://www.ncbi.nlm.nih.gov/bioproject/PRJEB88235) | [SAMEA115325124](https://www.ncbi.nlm.nih.gov/biosample/SAMEA115325124) | [ERX14248513](https://www.ncbi.nlm.nih.gov/sra/ERX14248513%5baccn%5d) | PacBio |
| *Penicillus capitatus* | Bryopsidales | N | Y | [PRJEB87011](https://www.ncbi.nlm.nih.gov/bioproject/PRJEB87011) | [SAMEA9196995](https://www.ncbi.nlm.nih.gov/biosample/SAMEA9196995) | [ERX14151531](https://www.ncbi.nlm.nih.gov/sra/ERX14151531%5baccn%5d) | PacBio |
| *Caulerpa prolifera* | Bryopsidales | N | Y | [PRJEB94584](https://www.ncbi.nlm.nih.gov/bioproject/PRJEB94584) | [SAMEA115125908](https://www.ncbi.nlm.nih.gov/biosample/SAMEA115125908) | [ERX14899944](https://www.ncbi.nlm.nih.gov/sra/ERX14899944%5baccn%5d) | PacBio |

* **Datasets consisting of triplicate runs from the same biological sample, typically Illumina datasets, were concatenated prior to MAG recovery. Y indicates Yes, and N indicates No.**

**Table S2. Quality assessment by CheckM2 of MAGs recovered from coenocytic algae.**

**Table S3. Taxonomic prediction by GTDB-Tk of MAGs recovered from coenocytic algae.**

**Table S4. Final taxonomic prediction by GTDB-Tk of MAGs recovered from coenocytic algae.**

**Table S5. Metabolic KEGG pathways of MAGs recovered from coenocytic and non-coenocytic algae.**

**Table S6. BGC distribution in MAGs recovered from coenocytic and non-coenocytic algae.**

**Table S7. PUL profiles of MAGs recovered from coenocytic and non-coenocytic algae.**

**Table S8. Statistical analysis of KEGG pathways in MAGs recovered from coenocytic and non-coenocytic algae.**
